# *N*-(2-Amino­phen­yl)-2-anilinobenzamide

**DOI:** 10.1107/S1600536809013014

**Published:** 2009-04-25

**Authors:** Kun Huang, Feng Huang, Da-Bin Qin

**Affiliations:** a School of Chemistry and Chemical Engineering, China West Normal University, Nanchong 637002, People’s Republic of China

## Abstract

In the title compound, C_19_H_17_N_3_O, the planes of the aromatic substituents attached to the benzamide moiety are almost perpendicular to one another, making a dihedral angle of 88.16 (7)°. The observed conformation of the mol­ecule is produced by an intra­molecular N—H⋯O hydrogen bond.

## Related literature

For the synthesis, see: Martín *et al.* (2006 ▶); Charton *et al.* (2006 ▶). For related structures, see: Yusof *et al.* (2003 ▶); Du *et al.* (2009 ▶).
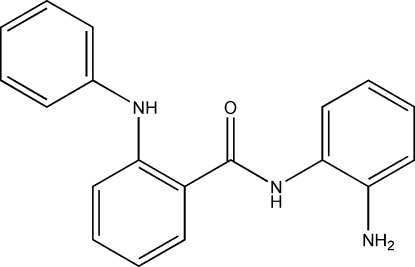


## Experimental

### 

#### Crystal data

C_19_H_17_N_3_O
*M* *_r_* = 303.36
Monoclinic, 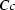

*a* = 6.707 (3) Å
*b* = 25.95 (1) Å
*c* = 9.480 (5) Å
β = 103.398 (7)°
*V* = 1605.0 (14) Å^3^
*Z* = 4
Mo *K*α radiation
μ = 0.08 mm^−1^
*T* = 93 K
0.40 × 0.27 × 0.10 mm

#### Data collection

Rigaku Spider diffractometer
Absorption correction: none
6515 measured reflections
1844 independent reflections
1695 reflections with *I* > 2σ(*I*)
*R*_int_ = 0.029

#### Refinement

*R*[*F* ^2^ > 2σ(*F* ^2^)] = 0.035
*wR*(*F* ^2^) = 0.072
*S* = 1.00
1844 reflections
224 parameters
2 restraints
H atoms treated by a mixture of independent and constrained refinement
Δρ_max_ = 0.14 e Å^−3^
Δρ_min_ = −0.15 e Å^−3^

### 

Data collection: *RAPID-AUTO* (Rigaku/MSC, 2004 ▶); cell refinement: *RAPID-AUTO*; data reduction: *RAPID-AUTO*; program(s) used to solve structure: *SHELXS97* (Sheldrick, 2008 ▶); program(s) used to refine structure: *SHELXL97* (Sheldrick, 2008 ▶); molecular graphics: *SHELXTL* (Sheldrick, 2008 ▶); software used to prepare material for publication: *SHELXTL*.

## Supplementary Material

Crystal structure: contains datablocks global, I. DOI: 10.1107/S1600536809013014/im2107sup1.cif

Structure factors: contains datablocks I. DOI: 10.1107/S1600536809013014/im2107Isup2.hkl

Additional supplementary materials:  crystallographic information; 3D view; checkCIF report

## Figures and Tables

**Table 1:** Hydrogen-bond geometry (Å, °)

*D*—H⋯*A*	*D*—H	H⋯*A*	*D*⋯*A*	*D*—H⋯*A*
N1—H1*N*⋯O1	0.88 (3)	1.96 (3)	2.714 (3)	142 (2)

**Fig. 1.**
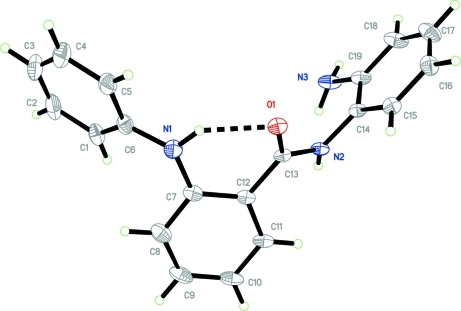
The molecular structure of the title compound, showing 30% probability displacement ellipsoids and the atomic numbering.

## supplementary crystallographic information

### Comment

Nowadays many researchers are intrested in the synthesis of new insecticides. Benzamide or its derivatives or analogs are used in the pharmaceutical industry for this purpose. We herein report the crystal structure of the title compound.

Bond lengths and angles in the title molecule (Fig. 1) are within normal ranges. The planes of the aromatic substituents attached to the benzamide moiety (C7—C12 and C14—C19) are almost perpendicular to one another, with a dihedral angle of 88.16 (7)° whereas the dihedral angle between C7—C12 and C1—C6 measures to 47.28 (9)°. The planes between C7—C12 and the amide moiety C12/C13/N2/O1 enclose an angle of 63.06 (8)°.

### Experimental

The title compound was prepared according to the reported procedure of Martín *et al.* (2006). and Charton *et al.* (2006). Colourless single crystals suitable for X-ray diffraction were obtained by recrystallization from dichloromethane.

### Refinement

H atoms were placed in calculated positions with N—H = 0.88–0.93 Å and C—H = 0.95 Å and refined using a riding model with *U*_iso_(H) = 1.2*U*_eq_(C,*N*). In the absence of significant anomalous dispersion effects, Friedel pairs were averaged.

### Crystal data

**Table:**

C_19_H_17_N_3_O	*F*(000) = 640
*M**_r_* = 303.36	*D*_x_ = 1.255 Mg m^−^^3^
Monoclinic, *C**c*	Mo *K*α radiation, λ = 0.71073 Å
Hall symbol: C -2yc	Cell parameters from 2858 reflections
*a* = 6.707 (3) Å	θ = 3.1–27.5°
*b* = 25.95 (1) Å	µ = 0.08 mm^−^^1^
*c* = 9.480 (5) Å	*T* = 93 K
β = 103.398 (7)°	Platelet, colorless
*V* = 1605.0 (14) Å^3^	0.40 × 0.27 × 0.10 mm
*Z* = 4	

### Data collection

**Table:**

Rigaku Spider diffractometer	1695 reflections with *I* > 2σ(*I*)
Radiation source: Rotating Anode	*R*_int_ = 0.029
graphite	θ_max_ = 27.5°, θ_min_ = 3.1°
ω scans	*h* = −8→8
6515 measured reflections	*k* = −33→31
1844 independent reflections	*l* = −12→12

### Refinement

**Table:**

Refinement on *F*^2^	Primary atom site location: structure-invariant direct methods
Least-squares matrix: full	Secondary atom site location: difference Fourier map
*R*[*F*^2^ > 2σ(*F*^2^)] = 0.035	Hydrogen site location: inferred from neighbouring sites
*wR*(*F*^2^) = 0.072	H atoms treated by a mixture of independent and constrained refinement
*S* = 1.00	*w* = 1/[σ^2^(*F*_o_^2^) + (0.0376*P*)^2^] where *P* = (*F*_o_^2^ + 2*F*_c_^2^)/3
1844 reflections	(Δ/σ)_max_ < 0.001
224 parameters	Δρ_max_ = 0.14 e Å^−^^3^
2 restraints	Δρ_min_ = −0.15 e Å^−^^3^

### Special details

**Table:**

Geometry. All e.s.d.'s (except the e.s.d. in the dihedral angle between two l.s. planes) are estimated using the full covariance matrix. The cell e.s.d.'s are taken into account individually in the estimation of e.s.d.'s in distances, angles and torsion angles; correlations between e.s.d.'s in cell parameters are only used when they are defined by crystal symmetry. An approximate (isotropic) treatment of cell e.s.d.'s is used for estimating e.s.d.'s involving l.s. planes.
Refinement. Refinement of *F*^2^ against ALL reflections. The weighted *R*-factor *wR* and goodness of fit *S* are based on *F*^2^, conventional *R*-factors *R* are based on *F*, with *F* set to zero for negative *F*^2^. The threshold expression of *F*^2^ > σ(*F*^2^) is used only for calculating *R*-factors(gt) *etc*. and is not relevant to the choice of reflections for refinement. *R*-factors based on *F*^2^ are statistically about twice as large as those based on *F*, and *R*- factors based on ALL data will be even larger.

### Fractional atomic coordinates and isotropic or equivalent isotropic displacement parameters (Å^2^)

**Table:**

	*x*	*y*	*z*	*U*_iso_*/*U*_eq_	
O1	0.4009 (2)	0.47225 (5)	0.56166 (13)	0.0295 (3)	
N2	0.3896 (2)	0.51617 (6)	0.35407 (16)	0.0257 (3)	
N1	0.4426 (3)	0.36962 (7)	0.52152 (19)	0.0368 (4)	
N3	0.0087 (3)	0.53595 (9)	0.16236 (19)	0.0418 (5)	
H3A	0.106 (4)	0.5228 (9)	0.121 (3)	0.053 (7)*	
H3B	−0.103 (4)	0.5495 (10)	0.097 (3)	0.063 (8)*	
C1	0.3021 (4)	0.28921 (8)	0.3998 (2)	0.0435 (6)	
H1	0.3647	0.2955	0.3212	0.052*	
C2	0.1803 (4)	0.24613 (9)	0.3980 (3)	0.0600 (7)	
H2	0.1605	0.2230	0.3183	0.072*	
C3	0.0878 (5)	0.23638 (10)	0.5100 (4)	0.0725 (9)	
H3	0.0028	0.2070	0.5080	0.087*	
C4	0.1209 (5)	0.27031 (10)	0.6262 (4)	0.0674 (8)	
H4	0.0588	0.2638	0.7049	0.081*	
C5	0.2422 (4)	0.31325 (9)	0.6295 (3)	0.0470 (6)	
H5	0.2633	0.3360	0.7100	0.056*	
C6	0.3335 (3)	0.32336 (8)	0.5156 (2)	0.0366 (5)	
C7	0.5729 (3)	0.38514 (8)	0.4348 (2)	0.0326 (5)	
C8	0.7016 (3)	0.34982 (9)	0.3887 (2)	0.0425 (5)	
H8	0.6954	0.3144	0.4127	0.051*	
C9	0.8377 (4)	0.36594 (10)	0.3087 (2)	0.0475 (6)	
H9	0.9231	0.3413	0.2773	0.057*	
C10	0.8519 (3)	0.41732 (10)	0.2733 (2)	0.0429 (6)	
H10	0.9467	0.4281	0.2187	0.052*	
C11	0.7264 (3)	0.45261 (9)	0.3184 (2)	0.0331 (5)	
H11	0.7366	0.4880	0.2952	0.040*	
C12	0.5841 (3)	0.43752 (8)	0.39754 (18)	0.0271 (4)	
C13	0.4524 (3)	0.47661 (7)	0.44460 (18)	0.0245 (4)	
C14	0.2603 (3)	0.55640 (7)	0.38352 (19)	0.0272 (4)	
C15	0.3209 (3)	0.58570 (8)	0.5089 (2)	0.0324 (4)	
H15	0.4505	0.5797	0.5728	0.039*	
C16	0.1931 (4)	0.62359 (9)	0.5410 (2)	0.0420 (5)	
H16	0.2341	0.6435	0.6269	0.050*	
C17	0.0059 (4)	0.63226 (10)	0.4473 (2)	0.0492 (6)	
H17	−0.0831	0.6579	0.4697	0.059*	
C18	−0.0532 (4)	0.60405 (10)	0.3216 (2)	0.0468 (6)	
H18	−0.1821	0.6108	0.2577	0.056*	
C19	0.0727 (3)	0.56574 (8)	0.2863 (2)	0.0339 (5)	
H1N	0.400 (4)	0.3966 (10)	0.563 (3)	0.053 (7)*	
H2N	0.414 (3)	0.5148 (7)	0.269 (2)	0.030 (5)*	

### Atomic displacement parameters (Å^2^)

**Table:**

	*U*^11^	*U*^22^	*U*^33^	*U*^12^	*U*^13^	*U*^23^
O1	0.0355 (7)	0.0417 (8)	0.0137 (6)	−0.0010 (6)	0.0106 (5)	−0.0004 (5)
N2	0.0232 (8)	0.0413 (9)	0.0144 (7)	0.0023 (7)	0.0076 (6)	0.0006 (7)
N1	0.0459 (11)	0.0375 (10)	0.0305 (9)	−0.0001 (8)	0.0162 (8)	−0.0020 (8)
N3	0.0251 (9)	0.0777 (15)	0.0209 (9)	0.0039 (9)	0.0022 (8)	−0.0025 (9)
C1	0.0528 (14)	0.0313 (11)	0.0418 (13)	0.0092 (10)	0.0019 (11)	0.0049 (10)
C2	0.0694 (18)	0.0288 (12)	0.0729 (18)	0.0060 (11)	−0.0017 (16)	0.0033 (12)
C3	0.078 (2)	0.0320 (14)	0.107 (3)	−0.0012 (13)	0.0214 (19)	0.0199 (16)
C4	0.084 (2)	0.0450 (15)	0.081 (2)	0.0042 (14)	0.0353 (17)	0.0261 (15)
C5	0.0580 (15)	0.0393 (12)	0.0475 (14)	0.0108 (11)	0.0199 (12)	0.0167 (11)
C6	0.0423 (13)	0.0324 (11)	0.0330 (11)	0.0081 (9)	0.0043 (9)	0.0101 (9)
C7	0.0315 (11)	0.0456 (13)	0.0207 (10)	0.0045 (9)	0.0058 (8)	−0.0018 (9)
C8	0.0457 (13)	0.0486 (13)	0.0325 (12)	0.0126 (10)	0.0076 (10)	−0.0025 (10)
C9	0.0411 (13)	0.0673 (17)	0.0364 (13)	0.0193 (12)	0.0137 (11)	−0.0061 (11)
C10	0.0275 (11)	0.0734 (17)	0.0294 (12)	0.0091 (10)	0.0098 (9)	−0.0053 (11)
C11	0.0226 (9)	0.0560 (13)	0.0207 (10)	0.0036 (9)	0.0050 (8)	−0.0017 (9)
C12	0.0239 (9)	0.0413 (11)	0.0151 (9)	0.0032 (8)	0.0025 (7)	−0.0017 (8)
C13	0.0198 (9)	0.0383 (11)	0.0153 (9)	−0.0034 (8)	0.0037 (7)	−0.0020 (8)
C14	0.0263 (10)	0.0371 (10)	0.0193 (9)	0.0006 (8)	0.0079 (8)	0.0041 (8)
C15	0.0366 (11)	0.0403 (11)	0.0211 (10)	−0.0017 (9)	0.0082 (8)	0.0019 (9)
C16	0.0592 (15)	0.0385 (12)	0.0340 (11)	0.0015 (10)	0.0225 (11)	0.0003 (10)
C17	0.0615 (16)	0.0546 (15)	0.0392 (14)	0.0227 (12)	0.0274 (13)	0.0120 (11)
C18	0.0390 (13)	0.0721 (16)	0.0319 (12)	0.0204 (12)	0.0132 (10)	0.0145 (11)
C19	0.0287 (11)	0.0548 (13)	0.0196 (9)	0.0035 (9)	0.0085 (8)	0.0064 (9)

### Geometric parameters (Å, °)

**Table:**

O1—C13	1.241 (2)	C7—C8	1.397 (3)
N2—C13	1.342 (2)	C7—C12	1.411 (3)
N2—C14	1.425 (2)	C8—C9	1.380 (3)
N2—H2N	0.86 (2)	C8—H8	0.9500
N1—C7	1.391 (3)	C9—C10	1.384 (4)
N1—C6	1.400 (3)	C9—H9	0.9500
N1—H1N	0.88 (3)	C10—C11	1.377 (3)
N3—C19	1.389 (3)	C10—H10	0.9500
N3—H3A	0.90 (3)	C11—C12	1.400 (3)
N3—H3B	0.93 (3)	C11—H11	0.9500
C1—C2	1.382 (4)	C12—C13	1.480 (2)
C1—C6	1.389 (3)	C14—C15	1.390 (3)
C1—H1	0.9500	C14—C19	1.398 (3)
C2—C3	1.372 (4)	C15—C16	1.384 (3)
C2—H2	0.9500	C15—H15	0.9500
C3—C4	1.387 (4)	C16—C17	1.378 (3)
C3—H3	0.9500	C16—H16	0.9500
C4—C5	1.376 (4)	C17—C18	1.376 (4)
C4—H4	0.9500	C17—H17	0.9500
C5—C6	1.383 (3)	C18—C19	1.394 (3)
C5—H5	0.9500	C18—H18	0.9500
			
C13—N2—C14	123.37 (15)	C8—C9—C10	121.1 (2)
C13—N2—H2N	118.0 (13)	C8—C9—H9	119.4
C14—N2—H2N	118.0 (13)	C10—C9—H9	119.4
C7—N1—C6	128.24 (19)	C11—C10—C9	119.0 (2)
C7—N1—H1N	110.2 (17)	C11—C10—H10	120.5
C6—N1—H1N	118.5 (17)	C9—C10—H10	120.5
C19—N3—H3A	117.9 (16)	C10—C11—C12	121.5 (2)
C19—N3—H3B	113.5 (16)	C10—C11—H11	119.3
H3A—N3—H3B	114 (2)	C12—C11—H11	119.3
C2—C1—C6	120.4 (2)	C11—C12—C7	119.10 (18)
C2—C1—H1	119.8	C11—C12—C13	119.91 (18)
C6—C1—H1	119.8	C7—C12—C13	120.96 (17)
C3—C2—C1	120.8 (3)	O1—C13—N2	122.03 (17)
C3—C2—H2	119.6	O1—C13—C12	121.07 (17)
C1—C2—H2	119.6	N2—C13—C12	116.89 (15)
C2—C3—C4	118.7 (3)	C15—C14—C19	120.58 (18)
C2—C3—H3	120.7	C15—C14—N2	119.84 (17)
C4—C3—H3	120.7	C19—C14—N2	119.57 (17)
C5—C4—C3	121.1 (3)	C16—C15—C14	120.2 (2)
C5—C4—H4	119.4	C16—C15—H15	119.9
C3—C4—H4	119.4	C14—C15—H15	119.9
C4—C5—C6	120.1 (3)	C17—C16—C15	119.5 (2)
C4—C5—H5	119.9	C17—C16—H16	120.2
C6—C5—H5	119.9	C15—C16—H16	120.2
C5—C6—C1	118.9 (2)	C18—C17—C16	120.5 (2)
C5—C6—N1	116.9 (2)	C18—C17—H17	119.8
C1—C6—N1	124.09 (19)	C16—C17—H17	119.8
N1—C7—C8	120.88 (19)	C17—C18—C19	121.2 (2)
N1—C7—C12	120.29 (18)	C17—C18—H18	119.4
C8—C7—C12	118.77 (19)	C19—C18—H18	119.4
C9—C8—C7	120.6 (2)	N3—C19—C18	120.96 (19)
C9—C8—H8	119.7	N3—C19—C14	121.04 (19)
C7—C8—H8	119.7	C18—C19—C14	117.92 (19)
			
C6—C1—C2—C3	0.2 (4)	N1—C7—C12—C13	−2.4 (3)
C1—C2—C3—C4	−0.8 (4)	C8—C7—C12—C13	−179.51 (17)
C2—C3—C4—C5	0.6 (4)	C14—N2—C13—O1	0.2 (3)
C3—C4—C5—C6	0.1 (4)	C14—N2—C13—C12	179.19 (17)
C4—C5—C6—C1	−0.7 (4)	C11—C12—C13—O1	−145.63 (18)
C4—C5—C6—N1	175.5 (2)	C7—C12—C13—O1	32.5 (3)
C2—C1—C6—C5	0.5 (3)	C11—C12—C13—N2	35.3 (2)
C2—C1—C6—N1	−175.4 (2)	C7—C12—C13—N2	−146.50 (18)
C7—N1—C6—C5	169.1 (2)	C13—N2—C14—C15	57.6 (2)
C7—N1—C6—C1	−14.9 (3)	C13—N2—C14—C19	−121.91 (19)
C6—N1—C7—C8	−37.1 (3)	C19—C14—C15—C16	1.8 (3)
C6—N1—C7—C12	145.9 (2)	N2—C14—C15—C16	−177.67 (18)
N1—C7—C8—C9	−176.8 (2)	C14—C15—C16—C17	−0.4 (3)
C12—C7—C8—C9	0.2 (3)	C15—C16—C17—C18	−0.9 (3)
C7—C8—C9—C10	0.7 (3)	C16—C17—C18—C19	0.7 (3)
C8—C9—C10—C11	−0.5 (3)	C17—C18—C19—N3	177.3 (2)
C9—C10—C11—C12	−0.7 (3)	C17—C18—C19—C14	0.7 (3)
C10—C11—C12—C7	1.6 (3)	C15—C14—C19—N3	−178.58 (18)
C10—C11—C12—C13	179.76 (18)	N2—C14—C19—N3	0.9 (3)
N1—C7—C12—C11	175.75 (17)	C15—C14—C19—C18	−2.0 (3)
C8—C7—C12—C11	−1.3 (3)	N2—C14—C19—C18	177.54 (18)

### Hydrogen-bond geometry (Å, °)

**Table:**

*D*—H···*A*	*D*—H	H···*A*	*D*···*A*	*D*—H···*A*
N1—H1N···O1	0.88 (3)	1.96 (3)	2.714 (3)	142 (2)
